# Supplementary material for: Cell cycle arrest enhances CD8+ T cell effector function by potentiating glucose metabolism and IL-2 signaling
Source: Nat Immunol. 2026 Jan 19;27(3):463–75. doi: 10.1038/s41590-025-02407-0 (PMC12956598; doi:10.1038/s41590-025-02407-0)
Supplement: Supplementary file 1 — Supplementary Figs. 1 and 2. [file 41590_2025_2407_MOESM1_ESM.pdf]

nature immunology

Supplementary information https://doi.org/10.1038/s41590-025-02407-0

Cell cycle arrest enhances CD8$^+$ T cell effector function by potentiating glucose metabolism and IL-2 signaling

In the format provided by the authors and unedited

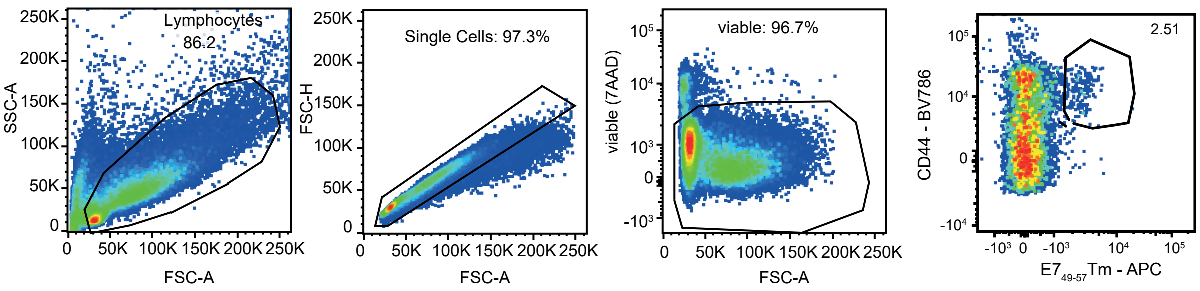

Supplementary Fig 1. Representative gating strategy for viable cells and $E7_{49\text{-}57}$-specific $CD8^+$ T cells. Flow cytometry plots showing the gating steps used to identify live cells and $E7_{49\text{-}57}$-specific $CD8^+$ T cells identfied by MHC class I tetramers.

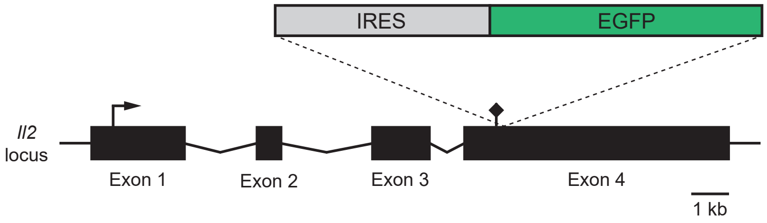

Supplementary Fig. 2. Generation of IL-2^{GFP} reporter mice.
IL-2^{GFP} reporter mice (B6.Il2em1Lumc; MGI:6452432) were generated in collaboration with the Transgenesis Facility Leiden of the LUMC by inserting a targeting construct encoding an internal ribosome entry site (IRES) and the fluorescent protein eGFP into the endogenous Il2 locus. Insertion was achieved via homologous recombination, following CRISPR/Cas9-mediated targeting of the Il2 locus (guide sequence GAGCCTTATGTGTTGTAAGC) in murine embryonic stem (ES) cells, resulting in replacement of the Il2 stop codon and 3'untranslated region (UTR) sequences of exon 4 (crRNA, tcrRNA and Cas9 protein obtained from IDT). Correctly targeted ES cell clones were identified by PCR and sanger sequencing, and injected into C57Bl/6J blastocysts to generate chimeric mice. PCR and sanger sequencing analysis of four predicted off-target sites (based CRISPOR.tefor.net scores) showed no off-targets events. Offspring of chimeric mice was bred with C57Bl/6J mice to achieve germ line transmission of the reporter gene. Mice were maintained on a C57Bl/6J background.
