# Supplementary material for: Cell cycle arrest enhances CD8+ T cell effector function by potentiating glucose metabolism and IL-2 signaling
Source: Nat Immunol. 2026 Jan 19;27(3):463–75. doi: 10.1038/s41590-025-02407-0 (PMC12956598; doi:10.1038/s41590-025-02407-0)
Supplement: Supplementary file 2 — Reporting Summary [file 41590_2025_2407_MOESM2_ESM.pdf]

nature portfolio

Corresponding author(s): Ramon Arens

Last updated by author(s): Nov 28, 2025

Reporting Summary

Nature Portfolio wishes to improve the reproducibility of the work that we publish. This form provides structure for consistency and transparency in reporting. For further information on Nature Portfolio policies, see our Editorial Policies and the Editorial Policy Checklist.

Statistics

For all statistical analyses, confirm that the following items are present in the figure legend, table legend, main text, or Methods section.

n/a	Confirmed	
☐	☒	The exact sample size (n) for each experimental group/condition, given as a discrete number and unit of measurement
☐	☒	A statement on whether measurements were taken from distinct samples or whether the same sample was measured repeatedly
☐	☒	The statistical test(s) used AND whether they are one- or two-sided<br>*Only common tests should be described solely by name; describe more complex techniques in the Methods section.*
☒	☐	A description of all covariates tested
☐	☒	A description of any assumptions or corrections, such as tests of normality and adjustment for multiple comparisons
☐	☒	A full description of the statistical parameters including central tendency (e.g. means) or other basic estimates (e.g. regression coefficient) AND variation (e.g. standard deviation) or associated estimates of uncertainty (e.g. confidence intervals)
☐	☒	For null hypothesis testing, the test statistic (e.g. F, t, r) with confidence intervals, effect sizes, degrees of freedom and P value noted<br>*Give P values as exact values whenever suitable.*
☒	☐	For Bayesian analysis, information on the choice of priors and Markov chain Monte Carlo settings
☒	☐	For hierarchical and complex designs, identification of the appropriate level for tests and full reporting of outcomes
☒	☐	Estimates of effect sizes (e.g. Cohen's d, Pearson's r), indicating how they were calculated

Our web collection on statistics for biologists contains articles on many of the points above.

Software and code

Policy information about availability of computer code

Data collection

BD FACSAria & BD Fortessa (BD Biosciences), Cytek Aurora (CYTEK), EVOS® FL Auto Imaging system (ThermoFisher), SpectraMax i3x Multi-Mode Microplate Reader (Molecular Devices), AMERSHAM Imager 600 (Bimedis), Novaseq 6000 (Illumina), General Metabolics' high-throughput non-targeted metabolomics platform (consisting of an Agilent 1260 Infinity II LC pump coupled to a Gerstel MPS autosampler (CTC Analytics, Zwingen, Switzerland) and an Agilent 6550 Series Quadrupole TOF mass spectrometer (Agilent, Santa Clara, CA, USA) with Dual AJS ESI source operating in negative mode), Exploris480 mass spectrometer (Thermo), iMarkTM microplate reader (Biorad), Vectra system (Akoya Biosciences.

Data analysis

FlowJo (version 10), Morpheus software, OMIQ.ai, ImageJ, BIOWDL RNAseq pipeline v3.0.0, Ingenuity Pathway Analysis, Qlucore Omics Explorer (version 3.7), MATLAB, Graphpad Prism (version 10.2.3), QuPath (v0.3.1),RStudio (version 2024.04.2), R (version 4.4.1) and EdgeR (version 4.2.2).

Flow cytometry
Samples were arcsinh-transformed with relevant cofactors and then pre-gated on FSC/SSC, singlet, live, CD8+/CD3+/CD44+, after which 1842 of these gated cells were subsampled. Dimension-reduction was conducted using the UMAP algorithm (15 neighbors, minimum distance of 0.4). UMAPs were computed using the following markers: Sca-1, CD98, Glut1, Ki-67, CD25, CD127, PKM, CD43, KLRG1, Tetramer, and G6PD. For immune profiling of ex vivo CD8+ T cells, opt-SNE analysis was performed using the OMIQ.ai platform. Samples were arcsinh-transformed with relevant cofactors and then pre-gated on FSC/SSC, singlet, live, CD8+/CD3+, after which 545 of these gated cells were subsampled. Dimension-reduction was conducted using the opt-SNE algorithm (1000 iterations, perplexity 30, Theta 0.5 and random seed 4644) and computed using the following markers: CXCR3, PD-1, CD62L, CD25, LAG-3, CD127, and EOMES. Morpheus software (MA, USA, https://software.broadinstitute.org/morpheus) was used to create heatmaps. Tumor immune filtrates containing more than 5% B cells after perfusion were excluded from analysis.

RNA sequencing
The extracted RNA was converted into cDNA by using the SuperScript® III First-Strand Synthesis kit (Invitrogen). To block cDNA synthesis of ribosomal RNA, the extracted RNA was treated with QIAseq® FastSelectTM -rRNA HMR Removal Kit (Qiagen). Second strand DNA synthesis was performed by using the Klenow Fragment exo-polymerase kit (Invitrogen). IDT adapters containing an 8bp UMI sequence and enrichment was performed for the 150-250 bp fragments by using the Kapa Hyperprep Kit. The quality and concentration of the fragmented and enriched library was verified with the Agilent 2100 Bioanalyzer by using the High Sensitivity DNA kit (Agilent Technologies). PCR products were purified by using AmpureXP Beads (Beckman CoulterTM). First, pooling of the samples was checked by shallow sequencing on the MiSeq and afterwards the samples were run on the Novaseq 6000 (Illumina).
RNAseq reads were processed using the opensource BIOWDL RNAseq pipeline v3.0.0 (https://zenodo.org/records/3713261) developed at the LUMC. This pipeline performs FASTQ preprocessing (including quality control, quality trimming, and adapter clipping), alignment, read quantification, and optionally transcript assembly. FastQC (v0.11.7) was used for checking raw read QC. Adapter clipping was performed using Cutadapt (v2.4) with the default settings. RNAseq reads' alignment was performed using STAR (v2.7.3a) on mouse reference genome GRCm38. umi_tools (v0.5.5) was used to remove duplicates identified by UMIs.
Differential expression analysis on the datasets of Low et al.,(re-activation program of memory CD8 T cells), and the arrested versus released CD8+ T cells is done using RStudio (version 2024.04.2), R (version 4.4.1) and EdgeR (version 4.2.2). In EdgeR. the normalization method Trimmed-mean-of-M values "TMM" is used. Differentially Expressed Gene(s) (DEGs) are filtered with an FDR of < 0.05. The overlapping differentially expressed genes between both datasets (1519 genes) were visualized in a heatmap after per-gene z-scaling based on log2CPM of counts. Clustering is based on agglomerative hierarchical clustering using Euclidean distance (unsupervised), with four clusters.

Polar Metabolites
Mass spectrometry data processing and analysis were performed in MATLAB (The MathWorks, Natick, MA, USA) using functions from the Bioinformatics, Statistics, Database, and Parallel Computing toolboxes. Peak picking was performed once per sample on the total profile spectrum generated by summing all scans over time, using wavelet decomposition from the Bioinformatics toolbox. Peaks below 5,000 ion counts in the summed spectrum were excluded to avoid low-abundance features unlikely to yield meaningful insights. Centroid lists from individual samples were merged into a single matrix by binning accurate masses within instrument-specific resolution tolerances.
A list of expected ions, including deprotonated, fluorinated, and major adduct forms, was generated from HMDB v4.0. All molecular formulas matching the measured masses within 0.001 Da were enumerated. Because this method does not include chromatographic separation or extensive MS^2 characterization, compounds with identical molecular formulas cannot be distinguished; thus, annotation confidence corresponds to Level 4. In practice, confidence for primary metabolic intermediates is higher due to their abundance in biological extracts.
The resulting matrix contains the intensity of each mass peak in each sample, and a refined common m/z value was calculated using the weighted average of independently centroided values.

Phosphoproteomics data analysis and statistics
RAW files were searched against the Homo sapiens UniProt database (2023) using MaxQuant73 with default settings, adding Phospho(STY) as a variable modification and enabling FAIMS as appropriate. A maximum of four missed cleavages was allowed. Phosphosite intensities were imported into Perseus74, $\log_2$-transformed, and filtered to remove contaminants and reverse hits. Sites quantified in at least one sample per condition were retained, and missing values were imputed from a downshifted normal distribution (width $0.3\times\sigma$, downshift $1.8\times\sigma$). Z-scores were calculated after mean centering to normalize technical variation while preserving donor-specific biological differences. Student's t-test was applied to non-arrested and HU-released conditions, and responsive phosphosites were defined by $p < 0.05$ and >1.5-fold change.

For manuscripts utilizing custom algorithms or software that are central to the research but not yet described in published literature, software must be made available to editors and reviewers. We strongly encourage code deposition in a community repository (e.g. GitHub). See the Nature Portfolio guidelines for submitting code & software for further information.

Data

Policy information about availability of data

All manuscripts must include a data availability statement. This statement should provide the following information, where applicable:

- Accession codes, unique identifiers, or web links for publicly available datasets
- A description of any restrictions on data availability
- For clinical datasets or third party data, please ensure that the statement adheres to our policy

The mass spectrometry proteomics data have been deposited to the ProteomeXchange Consortium via the PRIDE partner repository with the dataset identifier PXD055517.
Reviewer account details:
Username: reviewer_pxd055517@ebi.ac.uk
Password: sXDvMdGjaUva

The RNAseq data have been deposited to the Gene Expression Omnibus (GEO).
To review GEO accession GSE277143:
Go to https://eur03.safelinks.protection.outlook.com/?url=https%3A%2F%2Fwww.ncbi.nlm.nih.gov%2Fgeo%2Fquery%2Facc.cgi%3Facc%3DGSE277143&data=05%7C02%7CT.C.van_der_sluis%40lumc.nl%7C6ba8084ee11d4c1ebbad08dcd88f2038%7Cc4048c4fdd544cbd80495457aacd2fb8%7C0%7C0%7C638623356452354578%7CUnknown%7CTWFpbGZsb3d8eyJWIjoiMC4wLjAwMDAiLCJQIjoiV2luMzIiLCJBTiI6Ik1haWwiLCJXVCI6Mn0%3D%7C0%7C%7C%7C&sdata=W1a%2FhMrrz1KWvjs5cacRwUAF9NYOnU8JSE8nXMMkXGo%3D&reserved=0
Enter token "wnurcqmetlcrbuj" into the box.

Research involving human participants, their data, or biological material

Policy information about studies with human participants or human data. See also policy information about sex, gender (identity/presentation), and sexual orientation and race, ethnicity and racism.

Reporting on sex and gender | Human PBMCs were isolated from buffy coats obtained from Sanquin (Amsterdam, Netherlands). Donors at Sanquin include

Reporting on sex and gender	all sexes and genders; samples were used without stratification. For the NEOLBC study, only female patients with breast cancer were included; all patient samples are therefore from females.
Reporting on race, ethnicity, or other socially relevant groupings	Human PBMCs were isolated from buffy coats obtained from Sanquin (Amsterdam, Netherlands). Donors at Sanquin include diverse races, ethnicities, and other socially relevant groupings; samples were used without stratification.
Population characteristics	Not applicable.
Recruitment	Recruitment criteria for the NEOLBC study are described in the corresponding manuscript (de Groot, Clin. Cancer Res. 2025).
Ethics oversight	The NEOLBC trial (NCT03283384) was conducted in accordance with the Declaration of Helsinki and approved by the Medical Ethical Committee of the LUMC.

Note that full information on the approval of the study protocol must also be provided in the manuscript.

Field-specific reporting

Please select the one below that is the best fit for your research. If you are not sure, read the appropriate sections before making your selection.

☒ Life sciences ☐ Behavioural & social sciences ☐ Ecological, evolutionary & environmental sciences

For a reference copy of the document with all sections, see nature.com/documents/nr-reporting-summary-flat.pdf

Life sciences study design

All studies must disclose on these points even when the disclosure is negative.

Sample size	Sample sizes for in vivo experiments (4–12 mice per group) were determined using G*Power or Power and Sample Size software and approved by the institutional statistician, providing 80% power at $\alpha = 0.05$. Sample sizes for ex vivo experiments were based on prior work in our laboratory to ensure sufficient numbers per group for informative results and statistical analysis.
Data exclusions	No animals or data points were excluded from the analyses, except one sample of the clinical data because the sample had insufficient cellularity (<25 cells/mm^2), which was below the predefined analysis threshold.
Replication	All experiments were independently repeated at least 2–3 times with similar results, including ex vivo CD8$^+$ T cell assays and in vivo tumor models. Representative images and flow plots illustrate typical outcomes. No findings failed replication. Reproducibility was supported by consistent statistical results in independent experiments and biological replicates.
Randomization	Mice were randomized before the start of each experiment, and in tumor studies they were additionally randomized based on tumor size. Male and female animals were matched for age and sex.
Blinding	Blinding was not performed for the in vivo experiments; however, for selected in vitro assays, the experimenter acquiring the data was blinded to treatment, yielding results consistent with the non-blinded experiments. The researcher responsible for staining and analyzing clinical samples was blinded to sample identity.

Reporting for specific materials, systems and methods

We require information from authors about some types of materials, experimental systems and methods used in many studies. Here, indicate whether each material, system or method listed is relevant to your study. If you are not sure if a list item applies to your research, read the appropriate section before selecting a response.

Materials & experimental systems

n/a	Involved in the study	
☐	☒	Antibodies
☐	☒	Eukaryotic cell lines
☒	☐	Palaeontology and archaeology
☐	☒	Animals and other organisms
☒	☐	Clinical data
☒	☐	Dual use research of concern
☒	☐	Plants

Methods

n/a	Involved in the study	
☒	☐	ChIP-seq
☐	☒	Flow cytometry
☒	☐	MRI-based neuroimaging

Antibodies

Antibodies used	Mouse Flow cytometry:<br>anti-CD122 APC; clone TM-b1 / Thermo Fisher / cat#17-1222-82 / RRID;AB_11151706

anti-CD127 Biotin; clone A7R34 / Thermo Fisher / cat#13-1271-85 / RRID;AB_466589
anti-CD127 RB780; clone A7R34 / BD Biosciences / cat#569066 / RRID;AB_3684748
anti-CD127 PE-Cy5; clone A7R34 / Biolegend / cat#135015 / RRID;AB_1937262
anti-CD25 PE/Cy7; clone PC61.5 / Thermo Fisher / cat#25-0251-82 / RRID;AB_469608
anti-CD25 PE; clone PC61 / BD Biosciences / cat#553866 / RRID;AB_395101
anti-CD25 APC; clone PC61.5 / Thermo Fisher / cat#17-0251-81 / RRID;AB_469365
anti-CD3 FITC; clone 145-2C11 / Thermo Fisher / cat#11-0031-85 / RRID;AB_464883
anti-CD3 PerCP-Cy5.5; clone 145-2C11 / Biolegend / cat#100327 / RRID;AB_893320
anti-CD3 BV785; clone 17A2 / Biolegend / cat#100231 / RRID;AB_11218805
anti-CD43 PE-Dazzle594; clone 1B11 / Biolegend / cat#121225 / RRID;AB_2687245
anti-CD44 IM7; clone BV785 / Biolegend / cat#103059 / RRID;AB_2571953
anti-CD44 eFluor450; clone IM7 / Thermo Fisher / cat#48-0441-82 / RRID;AB_1272246
anti-CD44 BUV805; clone IM7 / BD Biosciences / cat#741921 / RRID;AB_2871234
anti-CD45.1 PE; clone A20 / BD Biosciences / cat#553776 / RRID;AB_395044
anti-CD45.1 PE; clone A20 / BD Biosciences / cat#553776 / RRID;AB_395044
anti-CD62L PE; clone MEL-14 / BD Biosciences / cat#553151 / RRID;AB_394666
anti-CD69 eFluor450; clone H1.2F3 / Thermo Fisher / cat#48-0691-82 / RRID;AB_10719430
anti-CD8 FITC; clone 53-6.7 / Biolegend / cat#100706 / RRID;AB_312754
anti-CD8 APC; clone 53-6.7 / Biolegend / cat#100712 / RRID;AB_312751
anti-CD8 PerCP-Cy5.5; clone 53-6.7 / Biolegend / cat#100734 / RRID;AB_2075238
anti-CD8 APC-Fire750; clone 53-6.7 / Biolegend / cat#100766 / RRID;AB_2572113
anti-CD8 BUV395; clone 53-6.7 / BD Biosciences / cat#563786 / RRID;AB_2732919
anti-CD98 BUV615; clone RL388 / BD Biosciences / cat#752897 / RRID;AB_2916793
anti-CREL PE; clone 1RELAH5 / eBioscience / cat#12-6111-80 / RRID;AB_11042978
anti-CXCR3 PE; clone CXCR3-173 / Thermo Fisher / cat#12-1831-80 / RRID;AB_1210735
anti-EOMES PeCy7; clone Dan11mag / Thermo Fisher / cat#25-4875-82 / RRID;AB_2573454
anti-FDFT1 DyLight550; clone OT12F10 / Novus Biologicals / cat#NBP270715R / RRID;-
anti-G6PD APC-Cy7; clone EPR20668 / Abcam / cat#ab210702 / RRID;AB_2923527
anti-GLUT1 AF405; clone EPR3915 / Abcam / cat#ab252403 / RRID;AB_2783877
anti-Granzyme B PE; clone GB11 / Thermo Fisher / cat#12-8899-41 / RRID;AB_1659718
anti-ID2 eFluor450; clone ILCID2 / Thermo Fisher / cat#48-9475-82 / RRID;AB_2735053
anti-IFNγ APC; clone XMG1.2 / Thermo Fisher / cat#17-7311-82 / RRID;AB_469504
anti-IL-2 PE; clone JES6-5H4 / Thermo Fisher / cat#12-7021-82 / RRID;AB_466150
anti-Ki67 BV605; clone 16A8 / Biolegend / cat#652413 / RRID;AB_2562664
anti-KLRG1 BV786; clone 2F1 / Biolegend / cat#138429 / RRID;AB_2629749
anti-KLRG1 BV605; clone 2F1 / Biolegend / cat#138419 / RRID;AB_2563357
anti-KLRG1 PeCy7; clone 2F1 / Biolegend / cat#138416 / RRID;AB_2561736
anti-LAG3 PE; clone C9B7W / Biolegend / cat#125208 / RRID;AB_2133343
anti-NFATc1 AF488; clone 7A6 / Biolegend / cat#649603 / RRID;AB_2561822
anti-PD1 FITC; clone RMP1-30 / Thermo Fisher / cat#11-9981-82 / RRID;AB_465467
anti-PD1 BV605; clone 29F.1A12 / Biolegend / cat#135220 / RRID;AB_2562616
anti-PKM PE; clone EPR10138(B) / Abcam / cat#ab210448 / RRID;AB_2941747
anti-pS6 unconjugated; clone D68F8 / Cell Signaling Technology / cat#4858 / RRID;AB_916156
anti-pSTAT5 PE; clone SRBCZX / Thermo Fisher / cat#12-9010-42 / RRID;AB_2572671
anti-Sca-1 BUV496; clone D7 / BD Biosciences / cat#750169 / RRID;AB_2874374
anti-TNF FITC; clone MP6-XT22 / Biolegend / cat#506304 / RRID;AB_315425

Human Flow cytometry
anti-ACC1 PE-Cy7; clone EPR23235-147 / Abcam / cat#ab269273 / RRID;AB_3665505
anti-Aldolase AF647; clone EPR23181-39 / Abcam / cat#ab275162 / RRID;AB_3099487
anti-ATP5a DyLight488; clone EPR13030(B) / Abcam / cat#ab176569 / RRID;AB_2801536
anti-CD25 BV711; clone M-A251 / Biolegend / cat#356138 / RRID;AB_2632781
anti-CD25 BV421; clone M-A251 / BioLegend / cat#356113 / RRID;AB_2562163
anti-CD25 PE-Cy7; clone M-A251 / BD Biosciences / cat#557741 / RRID;AB_396847
anti-CD3 Amcyan; clone SK7 / BD Biosciences / cat#339197 / RRID;AB_647355
anti-CD3 AlexaFluor700; clone UCHT1 / BD Biosciences / cat#557943 / RRID;AB_396952
anti-CD8 Pacific Blue; clone RPA-T8 / BD Biosciences / cat#558207 / RRID;AB_397058
anti-CD8 PeCy7; clone SK1 / Biolegend / cat#344712 / RRID;AB_2044008
anti-CD8 FITC; clone SK1 / BioLegend / cat#344704 / RRID;AB_1877178
anti-CD8 AF488; clone SK1 / Biolegend / cat#344716 / RRID;AB_10549301
anti-CD8 APC; clone SK1 / Biolegend / cat#344722 / RRID;AB_2075388
anti-CD8 BUV805; clone SK1 / BD Biosciences / cat#612889 / RRID;AB_2833078
anti-CD8 APC-Cy7; clone SK1 / BD Biosciences / cat#348813 / RRID;AB_2868857
anti-CD98 BUV395; clone UM7F8 / BD Biosciences / cat#744508 / RRID;AB_2742283

Human + Mouse Flow cytometry (cross-reactive)
anti-CPT1a PE-Cy5; clone EPR21843-71-2F / Abcam / cat#ab137040 / RRID;AB_2884996
anti-FDFT1 DyLight550; clone OTI2F10 / Novus Biologicals / cat#NBP2-70715R / RRID; -
anti-G6PD APC-Cy7; clone EPR20668 / Abcam / cat#ab210702 / RRID;AB_2923527
anti-GLUT1 AF405; clone EPR3915 / Abcam / cat#ab252403 / RRID;AB_2783877
anti-PKM PE; clone EPR10138(B) / Abcam / cat#ab210448 / RRID;AB_2941747
anti-pS6 (Ser235/236) unconjugated; clone D68F8 / Cell Signaling Technology / cat#4858 / RRID;AB_916156
anti-SDHA AlexaFluor647; clone EPR9043(B) / Abcam / cat#ab310057 / RRID; -
anti-γH2AX (Ser139) PE; clone N1-431 / BD Biosciences / cat#562377 / RRID;AB_2737611

Human IF antibodies
anti-CD3 -1;1000 - labelled with Opal 540 (1;200); clone EPR449E / Abcam / cat#ab52959 / RRID;-
anti-CD8 - 1;500 - labelled with Opal 520 (1;100); clone D8A8Y / Cell Signaling Technology / cat#85336 / RRID;AB_2800052
anti-Glut1 - 1;2000 - labelled with Opal Opal 570 (1;400); clone EPR3915 / Abcam / cat#ab252403 / RRID;AB_10903230
anti-Purified anti-Pan-Cytokeratin - 1;2000 - labelled with Opal Antibody Opal 650 (1;800); clone AE1/AE3 / Biolegend / cat#914204 / RRID;AB_2616960

Additional antibodies:
anti-Foxk1 unconjugated; clone; - / Cell Signaling Technology / cat# 12025S / RRID;AB_2797801
anti-Anti-Histone H3 (tri methyl K9) unconjugated; clone polyclonal / abcam / cat#ab8898 / RRID;AB_306848
anti-α-Rabbit IgG (H+L) HRP; clone polyclonal / Thermo Fisher/Invitrogen / cat# G-21234 / RRID;AB_2536530

Viability Stain:
zombie aqua / Biolegend / cat#423102
zombie NIR / Biolegend / cat#423106
7-AAD / ThermoFisher/ cat#A1310

Validation

All commercially available antibodies have been validated by the respective suppliers. Anti-CD8 (clone 2.43), used for in vivo $CD8^+$ T-cell depletion, was validated by measuring the reduction of $CD8^+$ T cells in blood. Anti-PD-L1 (clone MIH-5), used for in vivo blockade of PD-1/PD-L1 interactions, was validated in a competition assay using fluorescently labeled MIH-5 for PD-L1 binding.

Eukaryotic cell lines

Policy information about cell lines and Sex and Gender in Research

Cell line source(s)

TC-1 (mouse lung, RRID: CVCL_4699). Obtained from T.C. Wu.
MC-38 (RRID:CVCL_B288).
(E.G7-OVA (RRID:CVCL_3505).
MIH5 hybridoma (MIH5 (RRID:CVCL_CW88). Obtained from M. Azuma.

Authentication

TC-1 was authenticated by the in vivo recognition of E7 specific CD8 T cells. MC-38 was authenticated by parallel experiments where T cells with multiple known MC-38 specificities were infiltrated in these tumors. E.G7-OVA was authenticated by the in vivo response to (activated) SIINFEKL specific CD8 T cells. MIH-5 antibodies obtained from hybridoma cultures were tested by flow cytometry in a competitive binding assay for PD-L1.

Mycoplasma contamination

All cell lines were routinely tested negative for Mycoplasma via PCR.

Commonly misidentified lines
(See ICLAC register)

The cell lines used are not reported as known misidentified cell lines by the International Cell line Authentication Committee.

Animals and other research organisms

Policy information about studies involving animals; ARRIVE guidelines recommended for reporting animal research, and Sex and Gender in Research

Laboratory animals

C57BL/6J mice were obtained from Charles River (L'Arbresle, France) and Janvier labs (Le Genest-Saint-Isle, France), and maintained in the animal facility of Leiden University Medical Centre (LUMC). OT-I mice (B6.Cg-PtprcaTg(TcraTcrb)1100Mjb), IL-2GFP-reporter mice (B6.Il2em1Lumc) (generated by the Transgenic Facility Leiden; Supplementary Fig. 2), IL-2flox/flox CreERT2)55, and Raptorflox/flox (B6.Cg-Rptortm2.1LexTg(Itgax-cre)1-1Reiz/J crossed in house with C57BL/6-Tg(Cd8a-cre)1Itan) were bred and maintained in the LUMC animal facility.

Animals were housed in individually ventilated cages under specific-pathogen free conditions at the animal facility at the Leiden University Medical Center (LUMC) at 20°C -22°C, a humidity of 45-65% RV and a light cycle of 6:30h-7:00h sunrise, 07:00h-18:00h daytime and 18:00h-18:30h sunset. All mice were 6-14 weeks of age. All animals received standard chow (SDS RM3 diet; DS801203G10R)

Wild animals

No wild animals were used in this study

Reporting on sex

Sex has not been considered in the design of the study.

Field-collected samples

The study does not involve samples collected in the field.

Ethics oversight

All animal experiments were approved by national (CCD) and local committees (Animal Welfare Body Leiden and Animal Experiment Committee Leiden) and performed under permit numbers AVD116002015271, AVD11600202013796, AVD11600202417987, and AVD1160020186804.

Note that full information on the approval of the study protocol must also be provided in the manuscript.

Plants

Seed stocks	NA
Novel plant genotypes	NA
Authentication	NA

Flow Cytometry

Plots

Confirm that:

- [x] The axis labels state the marker and fluorochrome used (e.g. CD4-FITC).
- [x] The axis scales are clearly visible. Include numbers along axes only for bottom left plot of group (a 'group' is an analysis of identical markers).
- [ ] All plots are contour plots with outliers or pseudocolor plots.
- [x] A numerical value for number of cells or percentage (with statistics) is provided.

Methodology

Sample preparation	Described in methods.
Instrument	Cytek Aurora (3L and 5L setup) and BD Fortessa flow cytometer (BD Biosciences) were used for analysis of cells. BD FACSAria was used for cell sorting for RNAsequencing.
Software	Flowjo software (TreeStar) and OMIQ data analysis software.
Cell population abundance	For RNA sequencing, spleens were isolated and a single cell suspension was obtained. CD8 T cells were enriched with magentic beads, CFSE labeled and stimulated as described in the methods. Purity of enriched cells was confirmed by flow cytometry. For sorting, lymphocytes were gated by FSC-A/SSC-A, then singlets were selected by FSC-A/FSC-H and SSC-A/SSC-H, exclusion of dead cells with 7AAD.
Gating strategy	For protein and or metabolic dye expression/intensity analysis, lymphocytes were gated by FSC-A/SSC-A, then singlets were selected by FSC-A/FSC-H, exclusion of dead cells by use of Zombie dyes or 7-AAD. Protein marker gating is based on the negative population from unstimulated cells.<br>Tetramer positive and KLRG1 postive cells were selected as followed: lymphocytes were gated by FSC-A/SSC-A, then singlets were selected by FSC-A/FSC-H, exclusion of dead cells by use of Zombie dyes or 7-AAD. Next cells CD3+/CD8+ cells were gated and Tetramer/CD44 double positive cells or KLRG1/CD44 double positive cells were gated and plotted. A gating strategy is provided as Supplementary Fig. 1.

- [x] Tick this box to confirm that a figure exemplifying the gating strategy is provided in the Supplementary Information.
