# Supplementary figures and images for: Cell cycle arrest enhances CD8+ T cell effector function by potentiating glucose metabolism and IL-2 signaling
Source: Nat Immunol. 2026 Jan 19;27(3):463–75. doi: 10.1038/s41590-025-02407-0 (PMC12956598; doi:10.1038/s41590-025-02407-0)

Fig. 5b

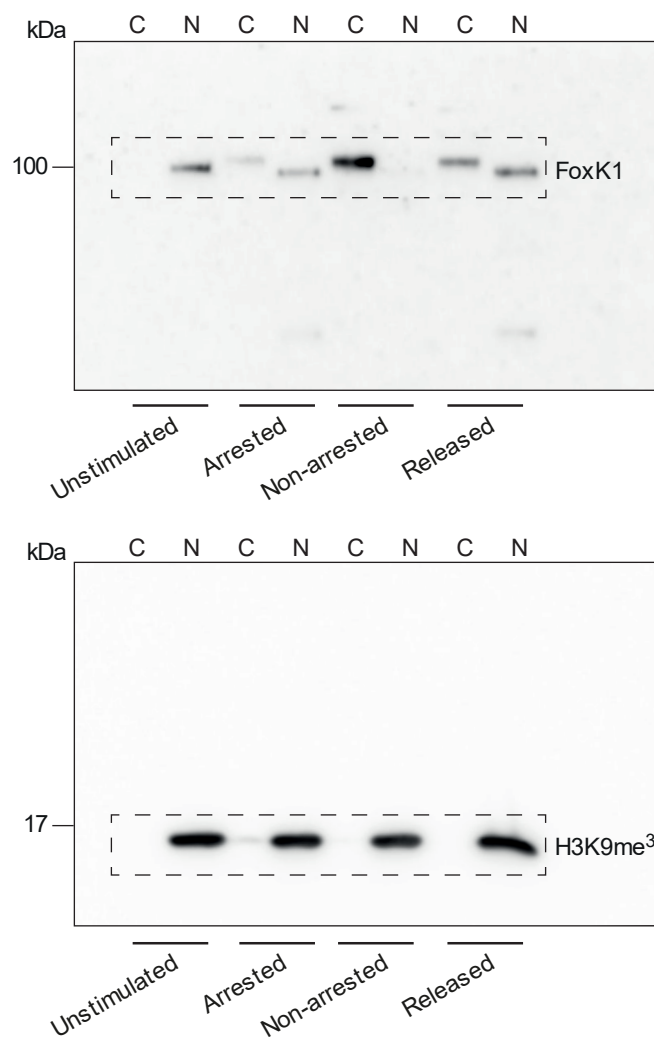

Supplement: Supplementary file 4 — Unprocessed western blots. [file 41590_2025_2407_MOESM4_ESM.pdf]
